# Reply: Phenotypic Characterization of Cardiac Involvement in Carriers With Pathogenic TTR Variants Warrants an Evidence-Based Approach

**DOI:** 10.1016/j.jacadv.2025.102318

**Published:** 2025-11-06

**Authors:** Naveen L. Pereira, Matteo Castrichini, Luca Fazzini

**Affiliations:** a Mayo Clinic, Rochester, Minnesota, USA; b University of Cagliari, Cagliari, Italy

We sincerely thank Prof Muller and colleagues for their thoughtful comments regarding our study on characterization of hereditary ATTRv amyloidosis in previously undiagnosed family members.^1^

We would like to clarify that, for the purposes of our paper, “previously undiagnosed” referred specifically to individuals who had not received a *clinical diagnosis* of cardiac amyloidosis at baseline. This does not imply absence of subclinical disease but rather reflects the clinical status at the time of enrollment.

The informative study by Muller et al.^2^ demonstrates that up to 75% of gene-positive relatives show no evidence of variant transthyretin amyloid cardiomyopathy (ATTR-CM) at baseline, even with bone scintigraphy and heart biopsy, and approximately 13% have no evidence of cardiac involvement by first-line testing (electrocardiography, echocardiography, and laboratory parameters). During a median follow-up of 3 years, only 9% developed heart failure, suggesting that most carriers, including those with ATTR-CM diagnosed at baseline, remained clinically stable within this time frame.

We agree that bone scintigraphy is an invaluable diagnostic modality and may reveal ATTR deposition before structural or functional abnormalities. However, the clinical significance of a positive pyrophosphate scan in the absence of significant echocardiographic changes or heart failure remains unclear. In our study, irrespective of whether cardiac amyloidosis was inferred by a positive scan, none of these individuals developed heart failure, and only about 10% demonstrated 2 or more echocardiographic features suggestive of ATTR-CM over a 6-year follow-up. These findings underscore that the natural history of subclinical ATTRv remains incompletely understood.

This uncertainty has important therapeutic implications. Our data raise the question of whether early therapy is justified solely based on positive scintigraphy or biopsy in asymptomatic individuals. Given the costs, potential side effects, and lifelong nature of treatment, careful assessment of risks and benefits is warranted. We share the view that validated, evidence-based algorithms for screening are essential to optimize early detection. At the same time, prospective longitudinal studies are needed to clarify the outcomes and prognosis of subclinical ATTRv and determine the threshold at which therapy meaningfully alters disease course. Ultimately, randomized trials—such as the Acoramidis Transthyretin Amyloidosis Prevention Trial (ACT-EARLY)^3^— will provide critical evidence to guide clinicians in balancing timely intervention with prudent stewardship of therapy.

We thank Prof Muller and colleagues for their insightful remarks. Their informative comments highlight the importance of ongoing dialogue and continued investigation to refine strategies for screening and management of transthyretin variant carriers.
